# Curcumin-Assisted Synthesis of MoS_2_ Nanoparticles as an Electron Transport Material in Perovskite Solar Cells

**DOI:** 10.3390/mi15070840

**Published:** 2024-06-28

**Authors:** Vajjiravel Murugesan, Balamurugan Rathinam

**Affiliations:** 1 Department of Chemistry, School of Physical and Chemical Sciences, B S Abdur Rahman Crescent Institute of Science and Technology, Chennai 600048, India; vajjiravel_m@crescent.education; 2 Department of Chemical and Materials Engineering, National Yunlin University of Science and Technology, 123 University Road, Section 3, Douliu, Yunlin 64002, Taiwan

**Keywords:** molybdenum disulfide, nanomaterials, perovskite solar cell, curcumin-derived organogel, sol–gel template, MoS_2_ nanoparticles

## Abstract

Recently, two-dimensional (2D) transition metal dichalcogenides (2D TMDs), such as molybdenum sulfide (MoS_2_) and molybdenum selenide (MoSe_2_), have been presented as effective materials for extracting the generated holes from perovskite layers. Thus, the work function of MoS_2_ can be tuned in a wide range from 3.5 to 4.8 eV by adjusting the number of layers, chemical composition, elemental doping, surface functionalization, and surface states, depending on the synthetic approach. In this proposed work, we attempt to synthesize MoS_2_ nanoparticles (NPs) from bulk MoS_2_ using two steps: (1) initial exfoliation of bulk MoS_2_ into few-layer MoS_2_ by using curcumin-cholesteryl-derived organogels (BCC-ED) and curcumin solution in ethylene diamine (C-ED) under sonication; (2) ultrasonication of the subsequently obtained few-layer MoS_2_ at 60–80 °C, followed by washing of the above chemicals. The initial treatment with the BCC-ED/C-ED undergoes exfoliation of bulk MoS_2_ resulted in few-layer MoS_2_, as evidenced by the morphological analysis using SEM. Further thinning or reduction of the size of the few-layer MoS_2_ by prolonged ultrasonication at 60–80 °C, followed by repeated washing with DMF, resulted in uniform nanoparticles (MoS_2_ NPs) with a size of ~10 nm, as evidenced by morphological analysis. Since BCC-ED and C-ED produced similar results, C-ED was utilized for further production of NPs over BCC-ED owing to the ease of removal of curcumin from the MoS_2_ NPs. Utilization of the above synthesized MoS_2_ NPs as an ETL layer in the cell structure FTO/ETL/perovskite absorber/spiro-OMeTAD/Ag enhanced the efficiency significantly. The results showed that MoS_2_ NPs as an ETL exhibited a power conversion efficiency (PEC) of 11.46%, a short-circuit current density of 18.65 mA/cm^2^, an open-circuit voltage of 1.05 V, and a fill factor of 58.66%, at the relative humidity of 70 ± 10% (open-air conditions) than that of the ED-treated MoS_2_ devices without curcumin. These results suggest that the synergistic effect of both curcumin and ED plays a critical role in obtaining high-quality MoS_2_ NPs, beneficial for efficient charge transport, lowering the crystal defect density/trap sites and reducing the charge recombination rate, thus, significantly enhancing the efficiency.

## 1. Introduction

Molybdenum disulfide (MoS_2_) has attracted increasing attention as one of the layered transition metal chalcogenides having S–Mo–S triatomic layers attached to each other via a weak van der Waals attraction force in the bulk. Two-dimensional MoS_2_ nanosheets exhibit excellent physical, chemical, optical, and electronic properties [1,2,3]. “Mo” atoms are sandwiched between two layers of “S” atoms, forming semiconducting 2H and metallic 1T phases. For example, unique properties of MoS_2_ include direct bandgap (~1.8 eV), good mobility (~700 cm^2^ V^−1^ s^−1^), high current on/off ratio of ~10^7^–10^8^, large optical absorption (∼10^7^ m^−1^ in the visible range), and a giant PL arising from the direct bandgap (1.8 eV) in the monolayer; which is distinct from its bulk counterpart (indirect band gap, ~1.2 eV) [4,5]. Thus, it has been studied widely for electronics and optoelectronics applications [1,6].

Despite its indirect bandgap, two to several layers of MoS_2_, or “few-layer MoS_2_”, still have attractive properties for various electronic devices because of its semiconductivity. However, the isolation of single-layer MoS_2_ and the study of the nature of this atomically thin film have clearly distinguished this mineral from other layered materials because of its unique properties, most notably semiconductivity and valley polarisation. The preparation of 2D MoS2 is, therefore, the first and the most crucial step in revealing and exploring its nature.

Many methods are reported for the isolation of single-layer MoS_2,_ including mechanical exfoliation [7], chemical exfoliation [5], the lithium intercalation method [8], vapor-phase growth, electron beam lithography [9]**,** etc. However, the drawbacks existing in each method, such as low yield and failure to grow large sheets of 2D MoS_2_, have led researchers to pursue the most promising chemical synthesis methods using molybdenum and sulphur precursors.

Among the large number of techniques recently developed to synthesize thin films and single/few-layer structures of MoS_2_, a versatile and industrially scalable one for large-scale production of MoS_2_ films is still in demand. For successful exfoliation, the solvation energy should circumvent the van der Waals attraction force between the layers in the bulk. In addition, the exfoliating solvent should have optimum material dispersity, polarity, and H-bonding components. Therefore, in this proposed work, we introduce a curcumin and ethylene diamine (ED)-assisted synthesis of MoS_2_ nanoparticles. Here, curcumin solution and organogels in ethylene diamine were selected for the exfoliation of MoS_2_. Curcumin and its derivatives have received considerable attention for their low toxicity and favorable potential in clinical applications, including antioxidant, anti-inflammatory, anti-tumor, anti-mutational, and anti-HIV properties [10,11]. In addition, curcumin can also be used as a sensor and reducing agent. Curcumin was reported as a reducing agent [12,13] for the reduction of Fe^3+^ to Fe^2+^ [12] as well as for AgNO_3_ [14]. The presence of the diketo moiety in the BCC structure seems to be essential both in redox reactions and in the scavenging of oxygen radicals [12]. In general, the organo/hydro gelators can be used as a template or support for the synthesis of nanoparticles such as silver nanoparticles (AgNP), gold nanoparticles (AuNPs), and iron nanoparticles (FeNPs). Since the gelators possess a 3D-network structure, a large number of functional groups can provide space for the nucleation and growth of the noble metal nanoparticles [15,16].

In our earlier report, bis-cholesteryl appended curcumin derivative (BCC), as an organogel in ED, was utilized for the synthesis of silver nanoparticles [17]. Therefore, we attempt to use BCC-ED gelators as template/structure directing agents for the synthesis of pure single/few-layer structures of MoS_2_. Moreover, a curcumin solution in ED (C-ED) and ED without the curcumin/curcumin derivative were also studied. The initial treatment using BCC-ED resulted in exfoliation into few-layer structures of MoS_2_. This may be due to the breaking of the intermolecular forces or bonds in between the layers of the bulk MoS_2_, leading to detaching the multi-layers into few-layers. However, further treatment with ultrasonication at heating resulted in nanoparticles because the initiation of the micromechanical acoustic cavitation process by ultrasonication produces microjets, shear forces, and shock waves, which cooperatively work on the breaking of detached layers into smaller crystals and, thus, lead to nanoparticles [18,19,20]. Similar results were observed for curcumin-ED (C-ED). Among these, C-ED was selected for further synthesis of MoS_2_-NPs, due to the ease of removal of curcumin from the MoS_2_-NPs. Thus, the obtained nanoparticles are highly pure, and the fabricated device using these high-quality MoS_2_-NPs enhances efficiency by 4.39% (synthesized without curcumin) to 11.46% (synthesized with curcumin). This method offers a low-temperature and uniform-size synthesis of high-quality MoS_2_ NPs in a shorter time than the reported high-temperature hydrothermal and sol–gel process [21,22,23]. The morphological and optical characterization of the synthesized MoS_2_ and their corresponding perovskite solar cells are discussed in detail with respect to the method of preparation of MoS_2_.

## 2. Materials and Methods

### 2.1. Materials

Fluorine-doped tin oxide (FTO) glass substrates (7 Ω·sq^−1^) were purchased from Ruilong optoelectronics (Taiwan). SnO_2_ colloidal solution (15%, Alfa Asear, Taiwan), formamidine hydroiodide (FAI, 98%, TCI, Taiwan), lead iodide (PbI2, 99.99%, TCI), methylamine hydrobromide (MABr, 98%, TCI), lead bromide (PbBr2, 98%, TCI), cesium iodide (CsI, 99.99%, Sigma-Aldrich, Taiwan), acetone (99.8%, AcroSeal, Taiwan), dimethylformamide (DMF, 99.8%, Sigma-Aldrich), dimethyl sulfoxide (DMSO, 99.9%, Sigma-Aldrich), methyl acetate (MA, 98%, Sigma-Aldrich), pentaerythritol triacrylate (PETA, Alfa Asear), 2-hydroxy-2-methylpropiophenone (HMPP, 97%, Alfa Asear), spiro-OMeTAD (99%, Sigma-Aldrich), lithium bis(trifluoromethyl-sulphonyl) imide (Li-TFSI, 99.95%, Sigma-Aldrich), FK209 Co(III) TFSI salt (FK209, 98%, Sigma-Aldrich, Taiwan), 4-tert-butylpyridine (4-tBP, 98%, Sigma-Aldrich, Taiwan), molybdenum sulfide (MoS_2_, Sigma-Aldrich, Taiwan), and acetonitrile (AN, 99.8%, Sigma-Aldrich, Taiwan) were used as received. All other chemicals, including curcumin, cholestery formate, and ethylene diamine, were purchased from Aldrich Chemicals (Taiwan) and were used without further purification.

### 2.2. Device Fabrication

**Figure 1:**
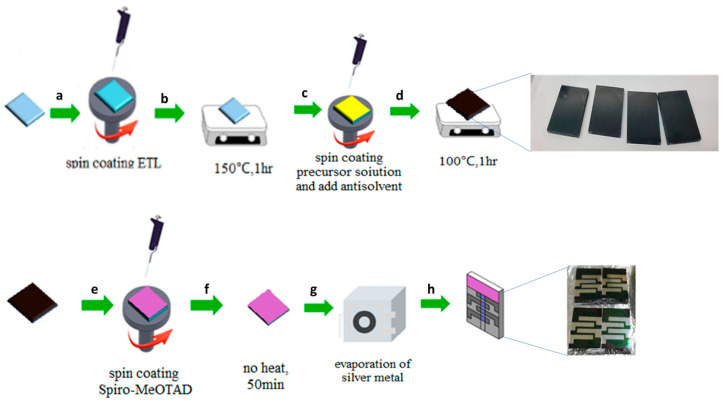
The schematic representation of fabrication of perovskite solar cells (**a**) deposition of MoS_2_; (**b**) annealing process; (**c**) coating of perovskite absorber; (**d**) annealing process; (**e**) doposition of hole trasport material; (**f**) drying process at room temperature, (**g**,**h**) coating of Silver as back contact.

The detailed fabrication process of PSCs is given in the Figure 1. Patterned FTO glass was sequentially cleaned with ethanol, isopropanol, and O_2_ plasma. Then, 100 µL of the electron transport layer solution (freshly prepared MoS_2_) was placed on the FTO glass evenly with a micropipette, spin-coated at 4000 rpm for 30 s, and annealed at 150 °C for an hour. The thickness of the spin-coated MoS_2_ (obtained from C-ED-2) film was found to be 63 to 68 nm based on the cross-sectional SEM images (given in the Result and Discussion section). CsI (0.087 M), FAI (1.47 M), MABr (0.23 M), PbI2 (1.47 M), and PbBr2 (0.23 M) were mixed in the mixture of DMF, DMSO, and acetone in a volume ratio of 7.2:1.8:1. The perovskite precursor solution (70 μL) was spin-coated onto the MoS_2_ substrates at 3000 rpm for 35 s in a chamber with approximately 70% RH. At the 12th s after the spin started, a solution of 0.05 vol.% MA (400 μL) was quickly dropped onto the coating layer. The sample was then annealed at 100 °C for 1 h. Then spiro-OMeTAD (72.3 mg), 4-tBP (28.8 μL), Li-TFSI (17.5 μL, 260 mg·mL^−1^ in AN), and FK209 (29 μL, 300 mg·mL^−1^ in AN) were mixed in 1 mL of chlorobenzene, and 70 μL of the Spiro-OMeTAD solution was spin-coated onto the top side of the perovskite layer at 2000 rpm for 30 s. Finally, a top electrode of 100 nm thick silver was deposited using thermal evaporation.

### 2.3. Synthesis of Bis-Cholesteryl Appended Curcumin (BCC)

BCC was synthesized as described in our earlier report and characterized spectroscopically [17]. Typically, about 1 g of curcumin (2.74 mmol) is placed in a 250 mL double neck round bottom flask and dissolved in dichloromethane (50 mL). To this solution, 0.66 g (5.429 mmol) of dimethyl aminopyridine (DMAP) is added and stirred well. To this mixture, the solution of cholesteryl chloroformate (3.65 g; 8.14 mmol) dissolved in dichloromethane is added dropwise over 30 min. The contents of the flask are stirred overnight with constant stirring at room temperature. After completion of the reaction, the contents of the flask are transferred to the separating funnel and washed with dil. HCl, aqueous sodium bicarbonate solution, and water, respectively. Then, dichloromethane was removed by distillation using a rotary evaporator. Thus, the obtained crude product was purified by washing with diethyl ether as well as re-precipitation in ethanol–water

FT-IR (KBr, νmax/cm^−1^): 3083 (CH=CH), 2944, 2827 (C-H), 1755 (COO), 1625 (C=O), 1465 (CH_2_ bending), 1378 (CH_3_ bending). ^1^H-NMR (CDCl_3_): 4.6 ppm (s, 2H, O=C-CH_2_-C=O), 7.5–7.7 (m, 2H, CH=CH), 7.1–7.4 ppm (m, 3H, Ar-H), 3.9 ppm (m, 8H, -O-CH_3_ and O-CH^−^), 5.4 (t, 2H, CH-CH_2_ in cholesteryl), 0.8–2.1 ppm (m, all methyl protons in cholesteryl unit) [17].

### 2.4. Curcumin-Assisted Synthesis of MoS_2_

**Figure 2:**
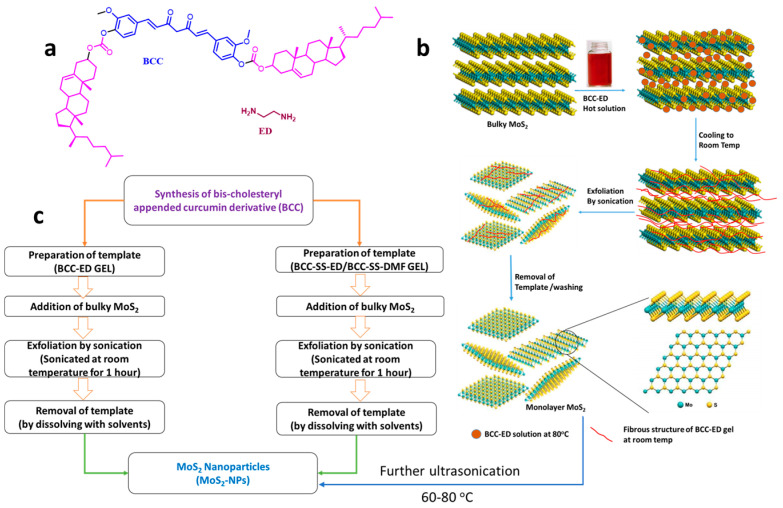
(**a**) Structure of the gel components, schematic representation of (**b**) exfoliation of MoS_2_ and then to NPs, and (**c**) process involved for the synthesis of high-quality MoS_2_ NPs.

The schematic representation of initial exfoliation of MoS_2_ to few/monolayer MoS_2_ using bis-cholesteryl-appended curcumin derivative (BCC) with ethylene diamine (BCC-ED) and further treatment under strong ultrasonication at higher temp, leading to NPs, are shown in the Figure 2.

The curcumin-based BCC-ED template was prepared by mixing 0.3 g of BCC with 10 mL of ethylene diamine (ED) and heated to obtain a clear solution, which was then allowed to cool for the formation of BCC-ED gel. Similarly, BCC-SS gels were prepared by adding 0.2 g of sodium stearate to 10 mL of dimethyl formamide (DMF), followed by a heating and cooling process, which leads to the formation of the gel.

About 0.2 g of bulky MoS_2_ is added to each gel and further subjected to sonication for 4 h. In addition, curcumin-ED solution was also prepared and utilized for the synthesis of MoS_2_. During the first 1 h of sonication, only large grains of MoS_2_ were observed. Therefore, sonication was continued for 4 h, with a 10 min break every hour.

The resulting solution was centrifuged, and the ED was removed by decantation process and then diluted with 10 mL of DMF. Thus, the obtained mixture was sonicated and then centrifuged at 1000 rpm for 5 min. The bulky MoS_2_ was removed and the upper solution was further centrifuged at 3000 rpm. The resulting solution was allowed to sonicate for 30 min in DMF and centrifuged at 6000 rpm for 15 min. In addition, instead of BCC, curcumin was mixed with ED and the resulting mixture was also used to break the bulky MoS_2_.

**Figure 3:**
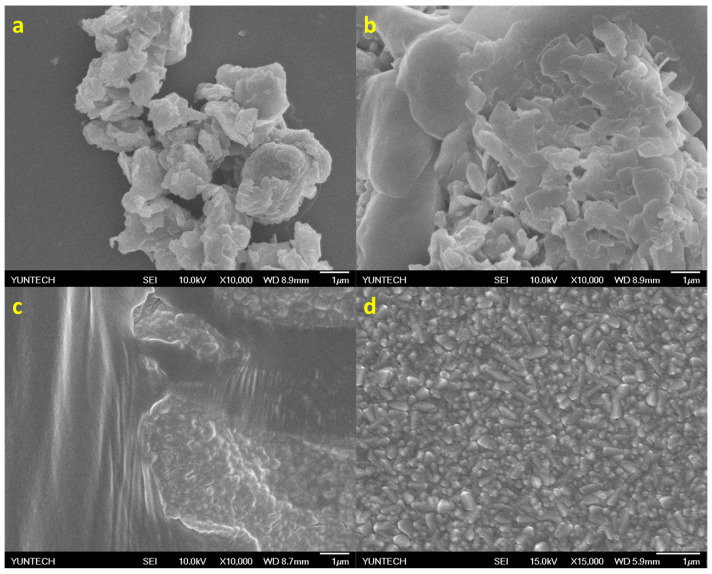
The SEM images of the exfoliated MoS_2_ (**a**) at initial exfoliation, (**b**) after 1 h of sonication with BCC-ED, (**c**) after 4 h of sonication with BCC-ED, and (**d**) after 4 h of sonication with curcumin-ED.

The BCC + ED and curcumin + ED exhibited similar results in the morphological analysis by scanning electron microscopy (SEM), as shown in Figure 3c,d. These results indicate that curcumin showed a synergistic effect with ED in the synthesis of MoS_2_. Moreover, complete removal of BCC was harder compared to curcumin. Therefore, curcumin mixed with ED was used for the successive synthesis of MoS_2_ NPs.

## 3. Results and Discussion

### 3.1. Morphological Analysis of MoS_2_ NPs

#### 3.1.1. Using BCC-ED Solution

The synthesized MoS_2_ were characterized by morphological analysis using SEM, and the morphologies were merged in Figure 3a–d. It was observed that after 1 h of sonication, separation/breaking of the multi-layers of MoS_2_ to few-layers occurred. Successive sonication with BCC-ED and curcumine-ED for 3 h resulted in a similar morphology (Figure 3c,d). The entangled fibrous structure of BCC-ED gels was observed in the BCC-ED exfoliated MoS_2_ (Figure 3c). However, pure MoS_2_ was observed in the curcumine-ED exfoliated MoS_2_. Moreover, the platelets or nanoparticles of MoS_2_ were in the size of a few nanometers (approximately 100 to 400 nm). This morphology is in accordance with the reported morphology of MoS_2_ in the literature [24,25], in which MoS_2_ was exfoliated using a high-temperature process, whereas we used a room-temperature process with the help of sonication, indicating the successful exfoliation of the MoS_2_.

#### 3.1.2. Using C-ED Solution

**Figure 4:**
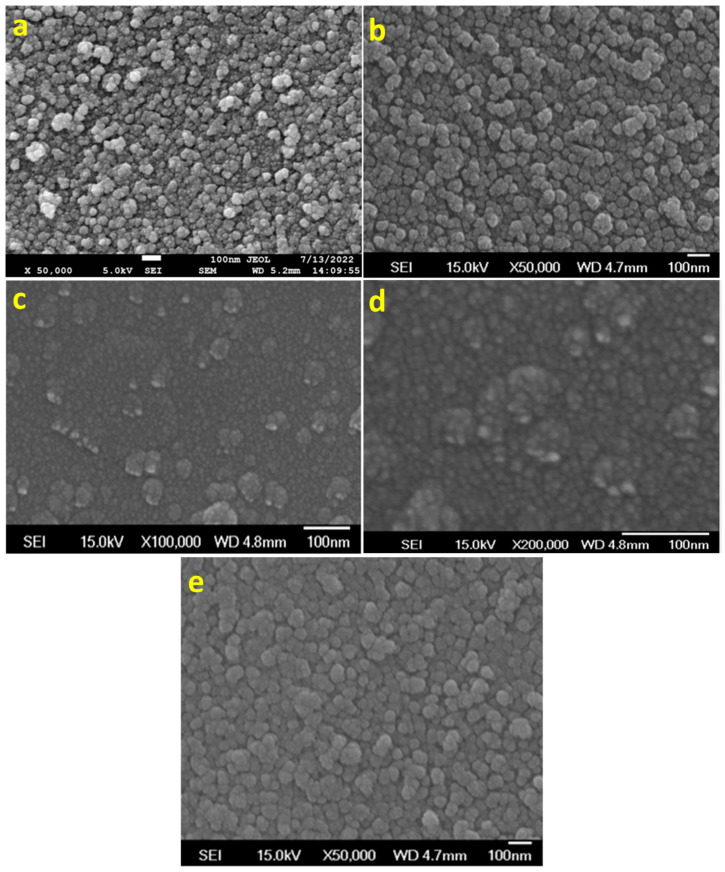
The morphological analysis of MoS_2_ prepared by using (**a**,**b**) ED, (**c**) C-ED-1, and (**d**) C-ED-2, and (**e**) different magnifications of (**d**).

Even though we successfully synthesized MoS_2_ NPs under room temperature with the help of ultrasonication, the efficiency of the obtained MoS_2_ as an ETL layer showed lower power conversion efficiency than expected. The morphological analysis of the MoS_2_-based perovskite layer by SEM analysis revealed rough surfaces with pin holes, which may lead to poor efficiency in the fabricated cells. The pin-hole formation may be due to two reasons. One might be the presence of curcumin (unremoved, if any), which may affect the high-quality film formation. Another might be from the various shapes of synthesized MoS_2_, as seen in Figure 3c,d, such as nanoparticles and nanorods, which affect the uniform coating of the ETL layer. In order to overcome these limitations, we try to reflux the curcumin–ethylene diamine solution containing MoS_2_ at 60–80 °C overnight, with constant stirring. For comparison, ethylene diamine was used alone without curcumin for the same purpose and then cooled to room temperature. A portion of the resulting solution was taken and centrifuged. The ED and C-ED were removed by decantation and washed with DMF. First-time washing (C-ED-1) resulted in a slightly brown-colored solution, indicating the existence of curcumin. Multiple/repeated washing with DMF resulted in a green-colored solution (C-ED-2). The morphological analysis of the curcumin–ethylene diamine (C-ED) assisted synthesis of MoS_2_ NPs is shown in Figure 4.

In the figure, rough surfaces with random-sized nanoparticles are observed for ED-treated MoS_2_ (Figure 4a). However, uniform particles are observed for C-ED-treated samples, indicating that curcumin plays an important role in the exfoliation and further breaking of MoS_2_ into NPs (Figure 4b). Hence, the agglomeration or clustering of nanoparticles was observed, which may be due to the presence of excess curcumin or ED as impurities or curcumin-induced aggregation [26,27,28]. The repeated washing with excess DMF and the ultrasonication process led to well-organized MoS_2_ nanoparticles with a size of approximately 10 nm being observed (Figure 4c–e), indicating the synergistic effect of curcumin and ethylene diamine [26,29]. Exfoliation of MoS_2_ to single or few-layered nanosheets is expected; however, instead, we observed nanoparticles in all the cases, which may be due to the prolonged ultrasonication causing the breaking of sheets into nanoparticles [1,30].

### 3.2. Fabrication of Perovskite Solar Cells Using Synthesized MoS_2_ NPs

In order to know the efficiency of the synthesized MoS_2_, it was utilized as electron transport material in the cell with the general structure of glass/FTO/ETL/perovskite absorber/spiro-OMeTAD/Ag, and their photophysical, optical, and power conversion efficiencies were studied. To gain insight into the film crystallinity, crystal size, and light-harvesting ability, the ED and C-ED-treated MoS_2_-based perovskite layers (FTO/MoS_2_/perovskite) were subjected to scanning electron microscopy (SEM), X-ray diffraction (XRD), and UV−vis absorption. The results are discussed below.

#### Photo-Physical Properties of the MoS_2_ NPs

**Figure 5:**
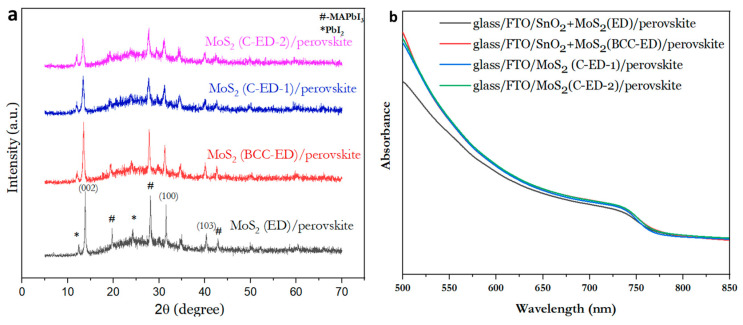
X-ray diffraction patterns (**a**) and the absorption spectra (**b**) of the perovskite layer consisting of MoS_2_ NPs, prepared at different conditions.

Figure 5a shows the XRD patterns of the glass/FTO/MoS_2_/perovskite. The peaks at 13.82°, 31.5°, and 40.39°, which correspond to the (002), (100), and (103) planes of the MoS_2_ nanosheet, respectively, and the diffraction pattern matched with the standard diffraction pattern of 2H–MoS_2_ [25]. The peaks at 19.7°, 23.8°, 28.1°, and 42.9° corresponded to the (200), (211), (220), and (314) planes for the perovskite, respectively [31]. However, the PbI_2_ (004), (008), and (0012) peaks located at 12.58°, 25.81°, and 35.5°, respectively, can be observed in all the samples. The coexistence of the two CH_3_NH_3_PbI_3_ and PbI_2_ phases was observed in the perovskite layers (CsFAMAPbI_3−x_Br_x_). This is due to the post-annealing process leading to the thermal decomposition of MAI and the formation of the PbI_2_ phase. In addition, (105), (110), and (008) planes of bulk MoS_2_ were absent, indicating the successful preparation of NPs [1].

Since the impurities could affect the light absorption of perovskite thin films due to the lower crystallite size in the perovskite layer, the light absorption of the perovskite thin films with different MoS_2_ were investigated through UV−vis spectroscopy and the results are given in Figure 5b. From the figure, it was observed that C-ED-treated MoS_2_ exhibited an absorption peak at ~750 nm, which is similar to the case of MoS_2_ treated with the BCC-ED template. These results suggest that the C-ED did not affect the light absorption properties of the perovskite layer. In addition, the C-ED-2 (multiple washing with DMF) showed better absorption than C-ED-1 (one-time washing), indicating that the presence of curcumin impurities affects the performance of the cell. Repeated washing leads to the complete removal of curcumin, ED, or any other impurities, which enhances the quality or crystal growth (large crystallites) in the perovskite layer. This can be attributed to the higher crystallinity of the high-quality perovskite film, supported by high-quality MoS_2_. All the spectrum shows a major peak around 740 nm. The results are in accordance with the reported literature [24], indicating the successful synthesis of MoS_2_.

In general, MoS_2_ can absorb visible light to some extent due to its 1.8 eV band gap and large optical absorption coefficient and, thus, has the possibility of affecting the light absorption of perovskite. However, the MoS_2_ can cover the visible spectrum only near to 400 nm, and it will not cover the entire region of the visible spectra (up to the wavelength of 700 nm) [32]. Moreover, once exfoliated, the magnitude of the peak for few-layer MoS_2_ is lower than multi-layer MoS_2_, which means multi-layer MoS_2_ absorbs light better than mono/few-layer MoS_2_ at 400 nm wavelength. In addition, MoS_2_ acts as a defect passivating layer, which somewhat lowers the charge carrier recombination. Therefore, exfoliated MoS_2_ is used as a dopant to passivate the metal surfaces such as Cu_2_O, Si_2_O, ZnO, etc. [33,34,35,36,37]. In our case, the MoS_2_ NPs enhanced the power conversion efficiency than the bulk or multi-layer MoS_2_, suggesting that the synthesized MoS_2_ NPs have very little effect or negligible effect than bulk towards the light absorption of perovskite.

**Figure 6:**
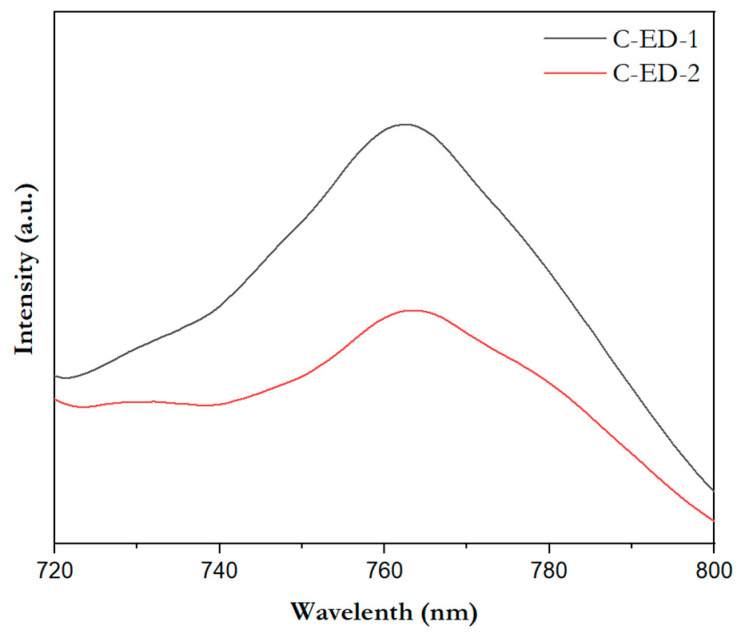
The emission spectra of the perovskite layer consisting of MoS_2_ prepared by C-ED with different washings.

To figure out the photoconversion process/charge transfer process associated with the quality of MoS_2_ in the perovskite films, the photoluminescence was recorded for the above said samples, and the results are merged in Figure 6. Here, the excitation wavelength of 400 nm is used, which is the specific excitation wavelength of perovskite (CsFAMAPbI_3−x_Br_x_). From the figure, it can be seen that the photoluminescence spectrum of perovskite generates energy around the emission wavelength of 750 nm. The PL intensity of the perovskite thin film sample prepared from the MoS_2_ ETL separated by C-ED-2 showed a significant decline than that of C-ED1, suggesting that C-ED-2 promotes electron extraction and transport capacity at the MoS_2_/perovskite interface through multiple interaction systems.

**Figure 7:**
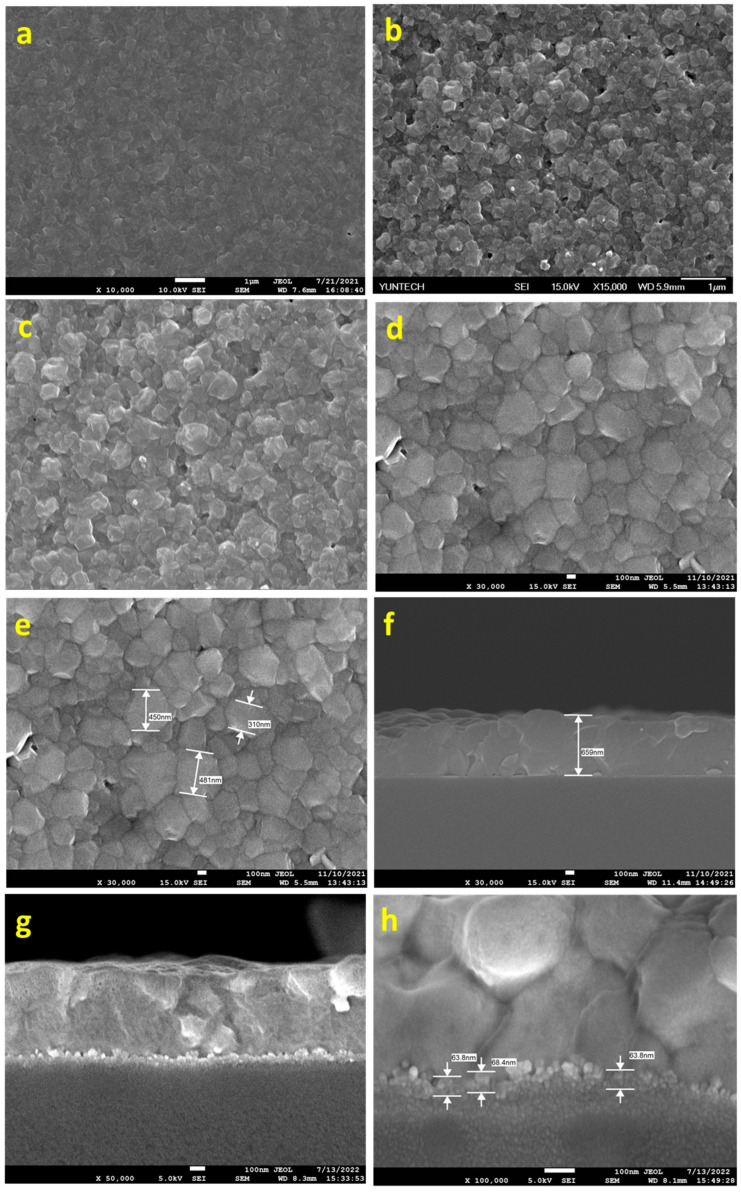
SEM morphology of perovskite layer consisting of different MoS_2_, (**a**) ED, (**b**) BCC-ED, (**c**) C-ED-1, (**d**,**e**) and C-ED-2, and (**f**) cross-sectional SEM image of Figure (**e**); (**g**,**h**) cross-sectional SEM image of the perovskites with MoS_2_ layer (C-ED-2) thickness.

The SEM morphology of the FTO/MoS_2_/perovskite were analyzed and merged in Figure 7. From the figure, it was observed that the morphology of the ED-treated MoS_2_-based device showed very small and random-sized perovskite crystals (Figure 7a). However, the BCC-ED-treated MoS_2_-based device showed better crystalline than that of ED (Figure 7b). But still seems to be low quality when compared with C-ED-treated devices (Figure 7c–e). It may be due to the unremovable BCC existing as impurities, which may affect the coating as well as the crystallization of perovskites.

Obviously, bigger-sized crystalline textures were observed for C-ED-2 than for C-ED-1, suggesting that curcumin interferes in the formation/crystallization of perovskites and, thus, needs to be washed thoroughly before being utilized as ETL in the fabrication PSCs. These results indicate that multiple washing (removal of curcumin) can effectively repair pin-hole issues. The cross-sectional SEM image of the C-ED-2-treated MoS_2_-based device is given in Figure 7f, which showed a thickness of 659 nm.

### 3.3. Photoconversion Efficiency of the Fabricated Devices

**Figure 8:**
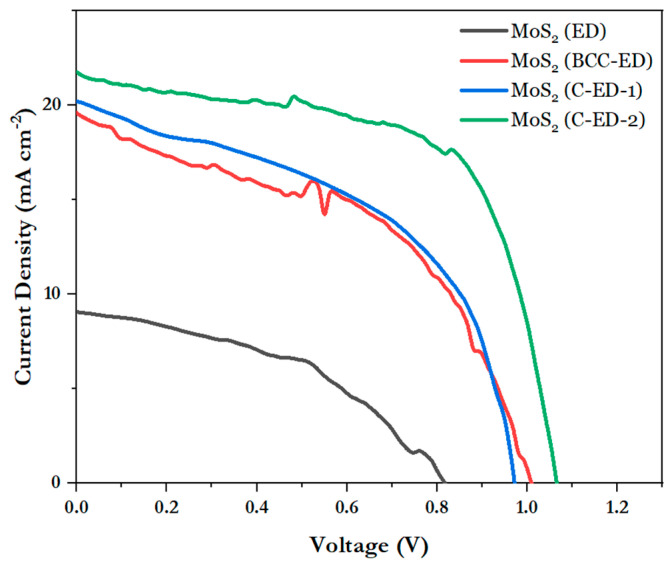
Photocurrent–voltage curves of perovskite solar cells with different electron transport layers.

The J-V curves of the perovskite solar cell with MoS_2_ as an electron transport material, prepared in different ways, are shown in Figure 8 and Table 1. It can be seen that ED-treated MoS_2_ solar cells exhibited low efficiency (4.39 ± 0.81) in the structure of FTO/ETL/perovskite/spiro-OMeTAD/Ag, and the curves were also very poor, which is due to the uneven particle size and the uneven surface of the electron transport layer, resulting in poor crystallization of the perovskite layer. Moreover, the MoS_2_ layer was not completely covered by the perovskite layer, and only moderate crystal growth of the perovskite layer was observed, as evidenced by SEM. Moreover, the surface of the perovskite layer was seemingly rough, with irregular packing of crystals, which may be due to the rough surface of the MoS_2_ layer. From the morphology of neat MoS_2_, it can be seen that the surface is not smooth, which may lead to poor packing of the perovskite layer, thus causing poor PCE. In the case of BCC-ED-treated MoS_2_-based devices, which exhibited better performance (PCE: 9.46) than ED, indicating that BCC improved the quality of the perovskite film by reducing the defects significantly. Interestingly, C-ED-treated MoS_2_ cells (C-ED-1 and C-ED-2) enhanced efficiency significantly because the smooth and uniform particles of MoS_2_ improved the quality of the thin film. Repeated washing or removal of excess curcumin improved the fill factor (FF) from 56 to 58%, as well as the open circuit voltage (V_oc_) from 0.94 to 1.05, revealing that C-ED-2 significantly enhanced the efficiency by reducing the recombination rate. This indicates that the quality of MoS_2_ plays a crucial role in the PCE of the devices. The high-quality MoS_2_ offers the best platform for perovskite film formation as well as a template for crystal growth, which is supported/consistent with SEM results. Therefore, any impurities like curcumin/ED that exist reduce the crystallinity of perovskite and increase the defects leading to poor efficiency. Because a larger crystal size also means lower crystal defect density and trap sites existing in the thin films, which is beneficial for efficient charge transport and reduced charge recombination. The study of the stability of the fabricated devices and other characterizations is in progress.

We also compared the efficiency of our synthesized MoS_2_ NPs with the reported MoS_2_ as ETL, shown in Table 2. From the table, it is observed that there are some reports that showed significantly higher efficiencies than our fabricated devices. However, these reported MoS_2_ were obtained by a high-temperature process or by using microwave or other commercially available methods. In addition, in some cases, MoS_2_ was used as a bilayer by combining it with other compounds such as graphene, TiO_2_, etc. Moreover, gold is used as the back contact in most of the reported structures, whereas silver is used in our fabricated devices. However, the efficiencies of our fabricated devices are comparable with the reported devices in the literature.

For further insights regarding the improved performance, especially the low voltage loss, the effect of MoS_2_ on the energy levels of the perovskite films was examined with ultraviolet photoemission spectroscopy (UPS). The UPS experiments probe the vacuum, Fermi, and valence energy levels near the perovskite layer, where electrons are extracted in the working solar cells. The value of the work function was calculated using the equation: WF = 21.2 − E_cut-off_ in which E_cut-off_ is the energy of emitted secondary electrons and 21.2 eV is according to the He laser UV light source [41].

**Figure 9:**
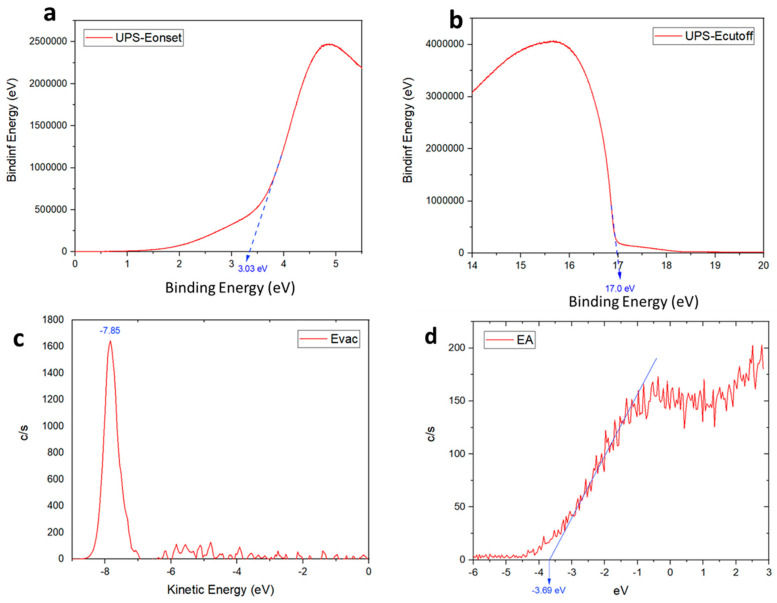
UPS spectra, (**a**) Fermi edge (EF), (**b**) E_cutoff_, and (**c**,**d**) LEIPS spectrum of MoS_2_ thin film (obtained from C-ED-2).

**Figure 10:**
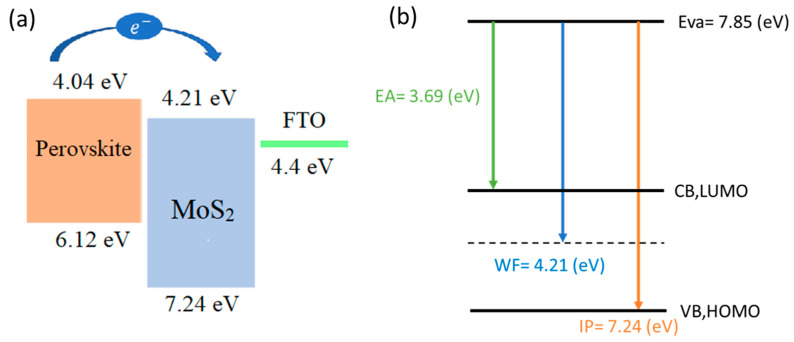
Schematic representation of (**a**) the energy-level diagram (**b**) HOMO-LUMO band diagram of the proposed MoS_2_.

The secondary electron cut-off energy of this material was 17.0 eV (Figure 9b). Therefore, the work function (WF) and valance band maximum (VBM) were calculated to be 4.21 eV and 7.24 eV, respectively. Additionally, from the low energy inverse photoelectron spectra (LEIPS), the vacuum energy level (EVAC) and electron affinity (EA) were obtained, and then conduction band maximum (CBM) could be calculated (EVAC-EA) as 4.16 eV. The energy differences between the perovskite and ETL layer is small, which is beneficial for charge transportation and enhanced efficiency. The energy-level scheme based on UPS is given in Figure 10.

## 4. Conclusions

In this study, the high-quality MoS_2_ were synthesized by using curcumin and ethylenediamine-based compounds C-ED and BCC-ED. The repeated washing with DMF (C-ED-2) under ultrasonication produced impurities (curcumin and ED) free high-quality nanoparticles with an average size of 10 nm as evidenced by the morphological analysis by SEM. Utilization of the C-ED-2-treated MoS_2_ nanoparticles as electron transport layer (ETL) in the cell structure FTO/ETL/perovskite absorber/spiro-OMeTAD/Ag enhanced the photoelectric conversion efficiency (PCE) from 4.39 to 11.46%, short-circuit current density increased from 9.3 mA/cm^2^ to 18.6 mA/cm^2^, open circuit voltage increased from 0.8 V to 1.05 V, and fill factor 57.4 to 58.6% that that of ED-treated MoS_2_ devices with a relative humidity of 70 ± 10%, indicating the synergistic effect of curcumin and ethylene diamine. These combinations cooperatively worked to synthesize high-quality MoS2 NPs, which facilitate the pin-hole free, large crystal growth of perovskite layers as supported by the light absorption and crystallinity and morphological analysis by using UV, PL, XRD and SEM. Therefore, such crystal growths enhanced the PCE significantly by lowering the crystal defect density and trap sites existing in the thin films, improving the efficient charge transport and thereby reducing charge recombination.

## Figures and Tables

**Table 1:** Photoelectric conversion efficiency of MoS_2_-based PVSCs with the basic structure of FTO/ETL/perovskite/spiro-OMeTAD/Ag.

MoS_2_ (from)	Voc (V)	Jsc (mA/cm^2^)	FF (%)	η (%)
ED	0.818	9.344	57.49	4.39 ± 0.81 (5.23)
BCC-ED	1.009	19.61	47.79	9.46 ± 0.58 (10.21)
C-ED-1	0.943	19.664	56.07	9.90 ± 1.03(10.32)
C-ED-2	1.05	18.65	58.66	11.46 ± 0.66 (12.13)

**Table 2:** Comparison of reported MoS_2_, cell structure, and efficiencies with respect to ETL.

Sl. No	MoS_2_ as ETL (Synthesized by)	Structure of PSCs	PCE (%)	Refs.
1	Microwave irradiation.	FTO/MoS_2_/perovskite/po-spiro-OMeTAD/Au	13.10	[24]
2	Hydrothermal method at 650 °C. MoS_2_ and triethylenetetramine-doped graphene (TETA-GR)-based bilayer used as ETL.	FTO/BL-(MoS_2_/TETA-GR)/MAPbI_3_/PTAA/Au	12.12	[38]
3	Electrospray deposited MoS_2_.	FTO/MoS_2_ nanosheets/perovskite/po-spiro-OMeTAD/Au	16.17	[39]
4	Commercially obtained as (CVD-MoS_2_ sheets with one-six layers uniformly grown on 300 nm SiO_2_/Si substrates).	Graphene/MoS_2_/MAPbI_3_/PTAA/Au	14.42	[40]
5	Ultrasonic spray pyrolysis method.	FTO/MoS_2_/perovskite/po-spiro-OMeTAD/Au	3.36	[25]
6	A liquid phase exfoliation technique employing N-Methyl-2-pyrrolidone (NMP) as solvent; TiO_2_/MoS_2_-based Nanocomposite (NC) as ETL.	ITO/NC(TiO_2_/MoS_2_)/MAPbI_3_/spiro-OMeTAD/Au	4.3	[36]
7	ZnO:MoS_2_ (0.5 wt %) composite layer as the ETL.	ITO/ZnO:MoS_2_/PTB7-TH:PC_71_BM/MoO_3_/Ag	10.1	[35]
8	C-ED-assisted synthesis at low temperature.	FTO/MoS_2_/perovskite/spiro-OMeTAD/Ag	11.46	This work

## Data Availability

The original contributions presented in the study are included in the article, further inquiries can be directed to the corresponding author.
